# Utility of Intraoperative Frozen Section in the Diagnosis of Periprosthetic Joint Infection

**DOI:** 10.1371/journal.pone.0102346

**Published:** 2014-07-15

**Authors:** Chuanlong Wu, Xinhua Qu, Yuanqing Mao, Huiwu Li, Kerong Dai, Fengxiang Liu, Zhenan Zhu

**Affiliations:** Department of Orthopaedics, Shanghai Ninth People's Hospital, Shanghai Jiaotong University School of Medicine, Shanghai, PR China; Stavanger University Hospital, Norway

## Abstract

**Purpose:**

Intraoperative frozen section (FS) is an effective diagnostic test for periprosthetic joint infection (PJI). We evaluated the diagnostic characteristics of single- and multiplex-site intraoperative FS, and evaluated the results of single-site FS combined with those of C-reactive protein (CRP) level and erythrocyte sedimentation rate (ESR) for assessing PJI.

**Methods:**

We studied 156 painful joint arthroplasties in 152 consecutive patients presenting for revision total joint arthroplasty due to PJI. Receiver operating characteristic analysis was used to determine the optimal cutoff values for CRP level, ESR, and intraoperative FS histopathology. Sensitivity, specificity, positive and negative predictive values, and accuracy of the diagnostic tests were assessed using a 2×2 table.

**Results:**

We investigated the diagnostic utility of polymorphonuclear leukocyte number (PMN) per high-power field (HPF) on FS. Our data showed that 5 PMNs per HPF is a suitable diagnostic threshold, with a high accuracy in single- and multiplex-site FS. Five PMNs in any 1 of 5 sites had the highest sensitivity of 0.86 and a specificity of 0.96. Five PMNs in every 1 of 5 sites had greater diagnostic utility, with a specificity of 1; however, the sensitivity of this measure fell to 0.62. Five PMNs in single-site FS had a sensitivity of 0.70 and a specificity of 0.94. Five PMNs in single-site FS or CRP level ≥15 mg/L increased the sensitivity to 0.92; however, the specificity decreased to 0.79.

**Conclusion:**

Compared with single-site FS, any 1 positive site on multiplex-site FS may improve sensitivity, while every 1 positive site on multiplex-site FS may improve specificity. Five PMNs in any 1 of 5 sites on FS has excellent utility for the diagnosis of PJI. Additional systematic large-scale studies are needed to verify this result.

## Introduction

Periprosthetic joint infection (PJI) is a critical complication of total joint arthroplasty. In the United States, PJI is the primary reason for total knee arthroplasty (TKA) failure and the third reason for total hip arthroplasty (THA) revision [1]. A multitude of tests are available for diagnosing PJI, including preoperative laboratory tests, plain radiographs, joint aspiration, and tissue culture [2]–[4]. However, the limited sensitivity and specificity of these tests pose difficulties in distinguishing between PJI and other causes of joint failure, such as aseptic loosening [5], [6]. Owing to its inexpensive cost, simple procedure, and timely report, intraoperative frozen section (FS) has become a promising and important tool for detecting PJI. Hospitals have successfully used intraoperative FS for diagnosing PJI, and recent reports have shown that this diagnostic method has a high accuracy compared with preoperative tests [7]–[10].

However, the optimal diagnostic thresholds of polymorphonuclear leukocyte number (PMN) per high-power field (HPF) on FS among studies are inconsistent, though many recommend 5 PMNs per HPF. A meta-analysis performed by Tsaras et al. showed that a threshold of 5 PMNs had a diagnostic odds ratio of 52.6, while a threshold of 10 PMNs had a diagnostic odds ratio of 69.8 [7]. In addition, few studies have evaluated the diagnostic utility of multiplex-site FS, which may increase the sensitivity and specificity of the diagnosis for PJI. Moreover, several studies have indicated that combined diagnostic methods may improve the detection of PJI, especially those that combine inflammatory laboratory markers. However, few studies have evaluated FS combined with laboratory tests, such as C-reactive protein (CRP) level and erythrocyte sedimentation rate (ESR), for the assessment of PJI.

Therefore, the purpose of this study was to evaluate the diagnostic test characteristics of single- and multiplex-site FS, and to evaluate the results of single-site FS combined with those of laboratory tests, including CRP level and ESR, for the assessment of PJI.

## Patients and Methods

### Ethics Statement

This study was a hospital-based investigation conducted at Shanghai Ninth People's Hospital in Shanghai, China. This study was approved by the Ethics Committee of Shanghai Ninth People's Hospital, and all participants provided written informed consent.

### Study Population

We systematically evaluated 177 painful joint arthroplasties in 173 consecutive patients who underwent revision surgery by 1 of 2 surgeons from January 2002 until May 2013. Of the 173 patients, 21 were excluded because of incomplete data, a preoperative diagnosis of inflammatory arthritis, or receiving antibiotics prior to the operation. A final total of 156 joint arthroplasties in 152 patients, including 140 hips and 16 knees, was available for evaluation. Four patients underwent revision of both joints.

### Diagnosis of PJI

A diagnosis of PJI was made, according to standard criteria, if at least 1 of the following was present: visible purulence surrounding the prosthesis or in the synovial fluid, acute inflammation on histopathologic examination of permanent tissue sections (as determined by the clinical pathologist), or positive culture results from 2 or more tissue or fluid samples obtained from the affected prosthesis or a sinus tract communicating with the prosthesis. Aseptic failure was defined as failure of the prosthesis in the absence of any of these criteria.

### Specimen Collection, FS Histologic Examination, and Laboratory Tests

Samples for histologic analysis were obtained from multiplex sites of periprosthetic membranes or tissue suspected to be infected. The FS of samples were studied using hematoxylin–eosin stain and based on Mirra's criteria (adapted by Feldman) [11], [12]. To avoid bias, all FS assessments were performed by 2 general pathologists who had no prior knowledge of the results of preoperative workup for infection. Multiple sections from each site were analyzed, and the number of PMNs per HPF (×400) was determined in at least 5 separate microscopic fields. Then, the average was calculated. FSs were examined and PMNs were counted using a OLYMPUS microscope (CX31) equipped with OLYMPUS objective lenses (×40 Plan C, field diameter 0.5 mm, numeric aperture 0.65, representing a total area of 0.1963 mm2 for one field of vision [13], [14]). Preoperative results of CRP level and ESR in peripheral blood were collected to assess the painful joints for PJI.

We evaluated the diagnostic utility of FS from 1, 3, 5, and 7 sites. We assessed the diagnostic utility of every 1 positive site on multiplex-site FS and any 1 positive site on multiplex-site FS for PJI. The optimal cutoff values for CRP level, ESR, and PMNs were tested by receiver operating characteristic curve(ROC) analysis. We analyzed the operating test when predetermined abnormal levels were used, assuming a diagnostic threshold for the diagnosis of PJI of 2 PMNs, 5 PMNs, and 10 PMNs per HPF on FS; ≥10 mg/L, ≥15 mg/L, and ≥20 mg/L for CRP level; and ≥20 mm/h, ≥30 mm/h, and ≥40 mm/h for ESR. We based these levels on our data as well as previously published criteria for the evaluation of PJI at the site of total joint arthroplasty [8], [11], [12], [15]–[18]. We made various comparisons between PMNs from single-site FS combined with CRP level or ESR. We considered “A or B” results as positive if at least 1 of the 2 tests was positive, and “A and B” results as positive if both tests were positive.

### Statistical Analysis

Baseline characteristics of the aseptic failure and PJI groups were compared using the Wilcoxon rank-sum or chi-square test, as appropriate. Sensitivity; specificity; positive predictive value; negative predictive value; and the accuracy of PMNs per HPF, CRP level, and ESR were based on a summary 2×2 table created for each test. A *P* value of less than 0.05 (for a 2-sided test) was considered statistically significant, and 95% confidence intervals were calculated as exact binomial confidence intervals. SPSS version 19.0 software was used to process other data.

ROC curves plotted with the use of MedCalc version 13.0 were used to examine the relationship between sensitivity and false-positive rate (100-specificity) based on the attributes of assignment into the infected or noninfected group. The area under the curve (AUC) was calculated for assessing diagnosis utility. An AUC of 1 demonstrates an ideal test with 100% sensitivity and 100% specificity, whereas an AUC of <0.5 indicates that the diagnostic test is less useful.

## Results

### Study Population

A total of 177 painful joint arthroplasties were identified, but 21 were later excluded owing to incomplete data or other reasons. Our final sample comprised 37 infection-related procedures (average age, 69.1 years; range, 41–86 years) and 119 noninfection-related procedures (average age, 63.9 years; range, 23–87 years).

Table 1 presents the reasons for the revision procedures. Persistent PJI was identified in 37 procedures (23.7%), while reasons for noninfection-related procedures included aseptic loosening (96 cases, 61.5%), periprosthetic osteolysis (11 cases, 7.1%), dislocation of the prosthesis (8 cases, 5.1%), shifting of the prosthesis (2 cases, 1.3%), and disjunction of the prosthesis (2 cases, 1.3%).

**Table 1:** Reason for revision procedures.

Preoperative Diagnosis	Number of Procedures
Aseptic loosening	96
Chronic infection	37
Periprosthetic osteolysis	11
Dislocation of the prosthetic	8
Shift of the prosthetic	2
Disjunction of the prosthetic	2
Total	156

Table 2 shows the basic characteristics of patients in the infection and noninfection groups, as well as the mean, range, and standard deviation of PMNs on FS and preoperative CRP level and ESR. The mean CRP level (64.4 vs. 8.8 mg/dL, *p*<0.001), ESR (53.8 vs. 26.8 mm/h, *p*<0.001), and PMNs (8.4 vs. 0.8, *p*<0.001) were significantly higher in patients with PJI than in those with noninfection-related failure.

**Table 2:** Characteristics of patients.

Classification	Infection	Non-infected	P Value†
Age (Mean, Range)	69.1 (41–86)	63.9 (23–87)	
Number of people (Male/female)	37 (18/19)	115 (55/60)	
Number of procedures (Hip/Knee)	37(26/11)	119(114/5)	
CRP (mg/dL)			<0.001
Mean	64.4	8.8	
Range	3.4–193	0.8–52.6	
Standard deviation	55.5	9.1	
Mean ESR (mm/h)			<0.001
Mean	53.8	26.8	
Range	2–109	2–101	
Standard deviation	32.8	25.2	
PMNs (per HPF)			<0.001
Mean	8.4	0.8	
Range	0.2–31.2	0–5.9	
Standard deviation	7.6	1	

CRP, C-reactive protein; ESR, erythrocyte sedimentation rate; PMNs, polymorphonuclear cells.

† Significance levels correspond to two-sided p values and were not adjusted for multiplicity.

### Threshold Value for FS

**Figure 1:**
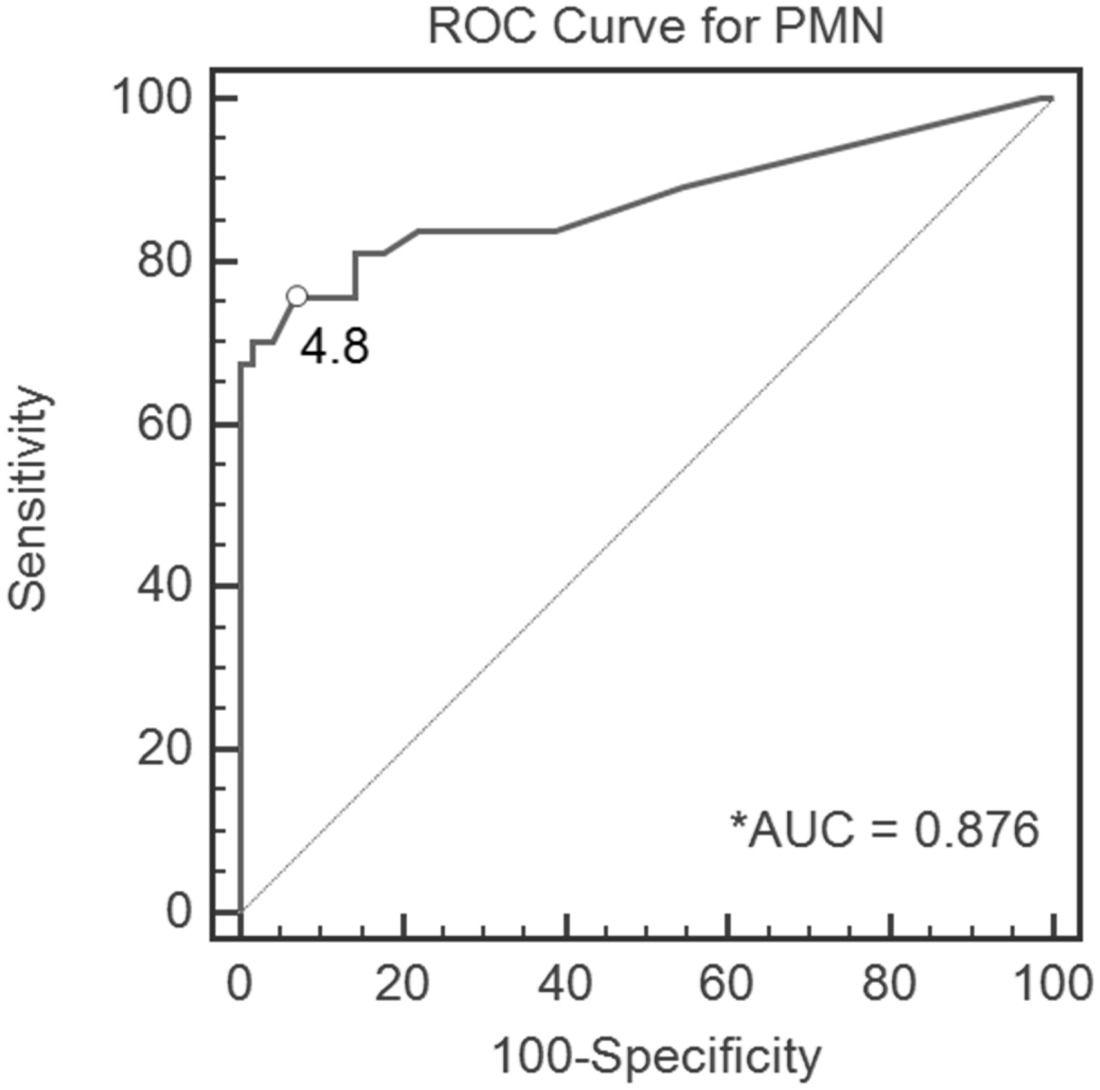
Receiver operating characteristic (ROC) curve, demonstrating the polymorphonuclear leukocyte number (PMN) cutoff value of 2.4 for all patients in the cohort. *AUC, area under the curve.

Table 3 examines the threshold value for FS. ROC analysis for all patients confirmed PMNs per HPF on single-site FS as a medium-quality diagnostic test (Fig. 1). The optimal PMN cutoff value for our entire patient cohort was 4.8. Since the difference between 4.8 and the standard of 5 is only 0.2, we set the appropriate threshold value as 5, while 2 PMNs per HPF and 10 PMNs per HPF also were studied. With the cut-off value set at 5, PMNs per HPF on single-site FS had a sensitivity of 0.70, specificity of 0.94, positive predictive value of 0.79, negative predictive value of 0.91, and accuracy of 0.88.

**Table 3:** Diagnostic test characteristics for frozen section.

Test	Infection	Noninfection	Sensitivity	Specificity	Positive predictive value	Negative predictive value	Accuracy
**Single site FS**	37	119					
PMNs≥2			0.78	0.86	0.63	0.93	0.84
PMNs≥5			0.70	0.94	0.79	0.91	0.88
PMNs≥10			0.30	1	1	0.82	0.83
**Every one positive of multiplex sites FS**							
**Three sites FS**	35	103					
PMNs≥2			0.73	0.93	0.77	0.92	0.88
PMNs≥5			0.68	0.97	0.86	0.91	0.90
PMNs≥10			0.27	1	1	0.82	0.83
**Five sites FS**	29	88					
PMNs≥2			0.68	0.97	0.86	0.91	0.90
PMNs≥5			0.62	1	1	0.89	0.91
PMNs≥10			0.27	1	1	0.82	0.83
**Seven sites FS**	15	31					
PMNs≥2			0.62	0.97	0.85	0.89	0.88
PMNs≥5			0.54	1	1	0.88	0.89
PMNs≥10			0.24	1	1	0.81	0.82
**Any one positive of multiplex sites FS**							
**Three sites FS**	35	103					
PMNs≥2			0.84	0.94	0.62	0.94	0.84
PMNs≥5			0.76	0.97	0.9	0.93	0.92
PMNs≥10			0.32	1	1	0.83	0.84
**Five sites FS**	29	88					
PMNs≥2			0.95	0.79	0.58	0.98	0.83
PMNs≥5			0.86	0.96	0.86	0.96	0.94
PMNs≥10			0.35	1	1	0.83	0.85
**Seven sites FS**	15	31					
PMNs≥2			1	0.78	0.59	1	0.83
PMNs≥5			0.86	0.92	0.78	0.97	0.91
PMNs≥10			0.35	1	1	0.83	0.85

CRP, C-reactive protein; ESR, erythrocyte sedimentation rate; FS, frozen section; PMNs, polymorphonuclear cells.

As the number of sites collected increased from 3 to 5 to 7, the sensitivity of 5 PMNs per HPF increased from 0.76 to 0.86 to 0.86, and the specificity dropped from 0.97 to 0.96 to 0.92 for any 1 positive site on multiplex-site FS; at the same time, the specificity of 5 PMNs per HPF increased from 0.97 to 1 to 1, and the sensitivity dropped from 0.68 to 0.62 to 0.54 for every 1 positive site on multiplex-site FS. In all, any 1 positive site in 5 sites had the highest accuracy of 0.94.

### Threshold Values for CRP Level and ESR

**Figure 2:**
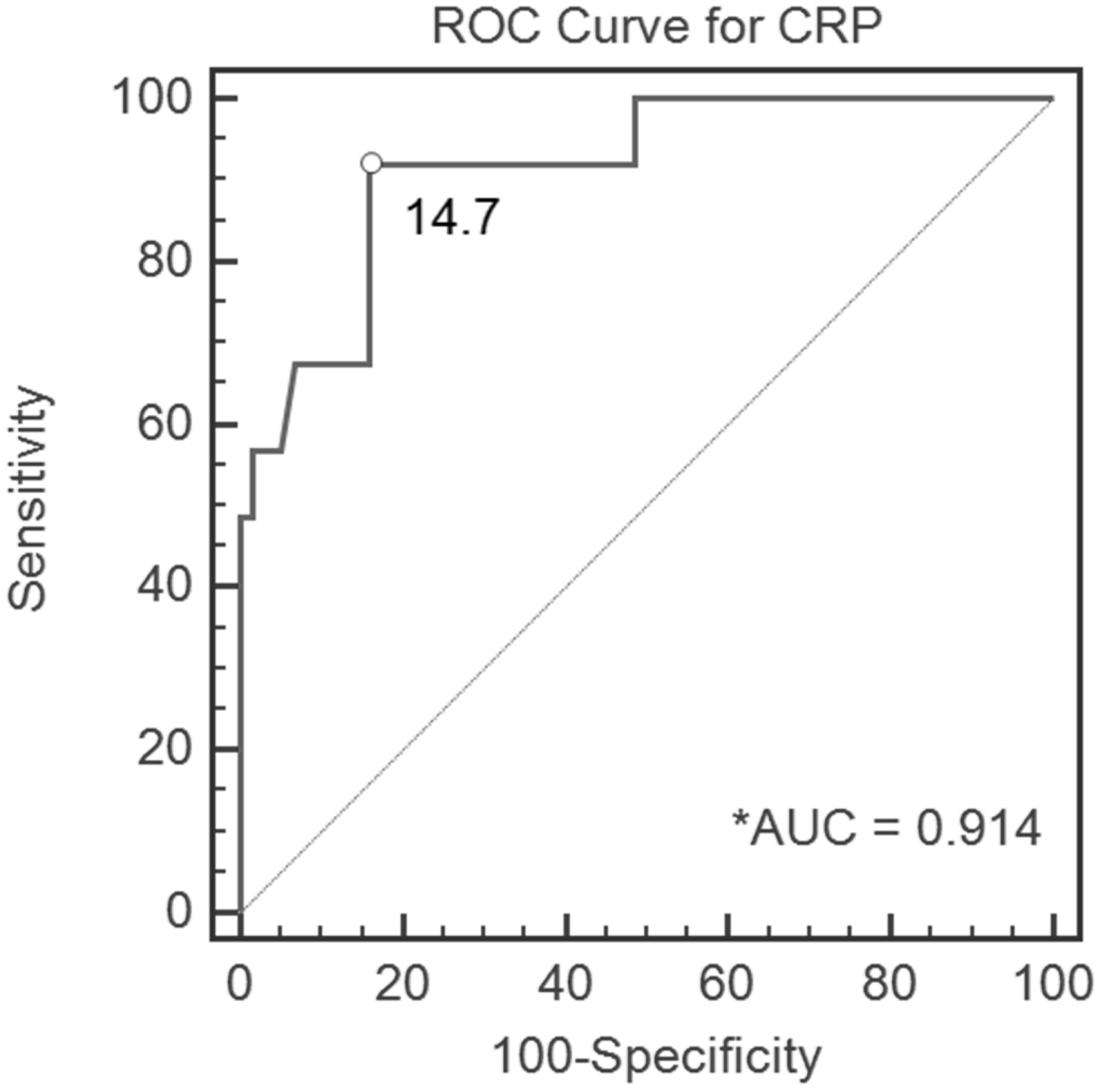
Receiver operating characteristic (ROC) curve, demonstrating the C-reactive protein (CRP) level cutoff value of 14.6 mg/L for all patients in the cohort. *AUC, area under the curve.

**Figure 3:**
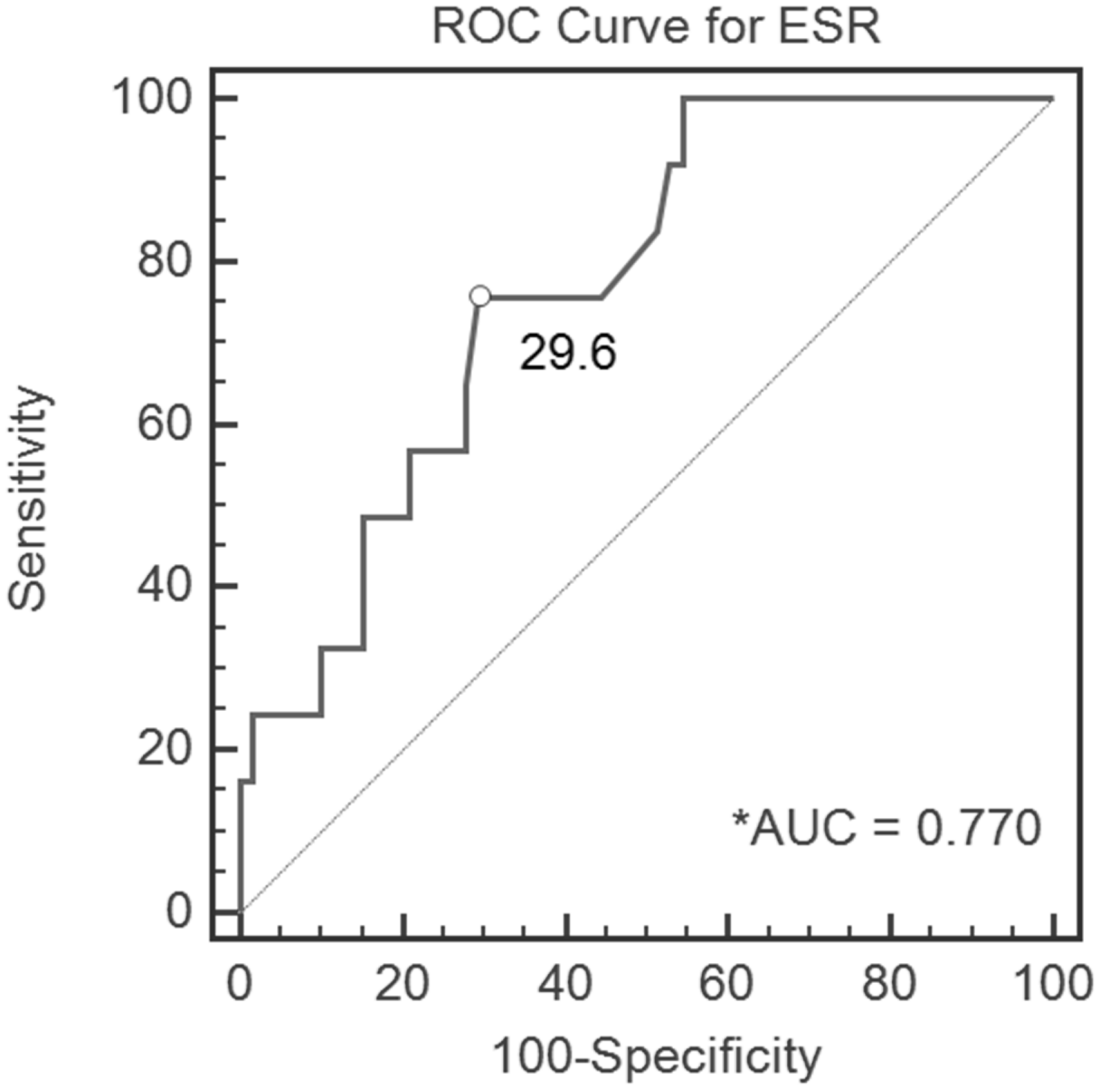
Receiver operating characteristic (ROC) curve, demonstrating the erythrocyte sedimentation rate (ESR) cutoff value of 28 mm/h for all patients in the cohort. *AUC, area under the curve.

Table 4 summarizes the threshold values for CRP level and ESR. ROC analysis demonstrated an optimal CRP level of 14.7 mg/L (Fig. 2) and an optimal ESR of 29.6 mm/h (Fig. 3). Ignoring the slight error, we set the appropriate CRP level threshold value as 15 mg/L and the ESR threshold value as 30 mm/h. According to the AUC, CRP level yielded a high-quality diagnostic value, with a sensitivity of 0.92, specificity of 0.84, and accuracy of 0.85 for CRP level ≥15 mg/L. ESR yielded a medium-quality diagnostic value, with a sensitivity of 0.75, specificity of 0.69, and accuracy of 0.7 for ESR≥30 mm/h.

**Table 4:** Diagnostic test characteristics for CRP, ESR, and combined tests.

Test	Sensitivity	Specificity	Positive predictive value	Negative predictive value	Accuracy
CRP≥10 mg/L	0.92	0.69	0.39	0.97	0.73
CRP≥15 mg/L	0.92	0.84	0.55	0.98	0.85
CRP≥20 mg/L	0.67	0.85	0.5	0.92	0.82
ESR≥20 mm/hr	0.92	0.45	0.27	0.96	0.54
ESR≥30 mm/hr	0.75	0.69	0.35	0.93	0.7
ESR≥40 mm/hr	0.58	0.78	0.37	0.9	0.75
PMNs≥5 (single site FS) or CRP≥15 mg/L	0.92	0.79	0.59	0.99	0.83
PMNs≥5(single site FS) and CRP≥15 mg/L	0.65	0.99	0.96	0.90	0.91
PMNs≥5(single site FS) or ESR≥30 mm/hr	0.92	0.65	0.40	0.96	0.71
PMNs≥5(single site FS) and ESR≥30 mm/hr	0.54	0.98	0.91	0.87	0.88

CRP, C-reactive protein; ESR, erythrocyte sedimentation rate; FS, frozen section; PMNs, polymorphonuclear cells.

### Diagnostic Value for Combined Test

Table 4 also describes the diagnostic value for the combined test. The combination of “5 PMNs in single-site FS AND CRP level ≥15 mg/L” increased the specificity of the test battery to 0.99, while the sensitivity decreased to 0.65. The combination of “5 PMNs in single-site FS OR CRP level ≥15 mg/L” demonstrated increased sensitivity (0.92) compared with that of either test alone, while specificity decreased to 0.79.

## Discussion

Recent clinical practice guidelines supported by the American Academy of Orthopaedic Surgeons state that FS is useful for ruling in PJI when such a diagnosis cannot be made preoperatively [19].The inconsistent diagnostic value for FS among studies interferes with clinical generalization, however. Moreover, few studies have evaluated the diagnostic utility of multiplex-site FS or combined tests, which may increase the sensitivity and specificity of the diagnosis for PJI. Our study attempted to provide evidence for the above-mentioned argument. Our data showed that 5 PMNs per HPF is a suitable diagnostic threshold, with a high accuracy in single- and multiplex-site FS. Five PMNs in single-site FS had a sensitivity of 0.70 and a specificity of 0.94. Five PMNs in any 1 of 5 sites had the highest sensitivity of 0.86 and a specificity of 0.96. Five PMNs in every 1 of 5 sites had greater diagnostic utility, with a specificity of 1; however, the sensitivity of this measure fell to 0.62. Five PMNs in single-site FS or CRP level ≥15 mg/L increased the sensitivity to 0.92; however, the specificity was 0.79.

### Utility of FS

FS seems to be the most reliable tool for intraoperative diagnosis and fits the conditions for an ideal test. One meta-analysis [7] indicated that intraoperative FS histologic evaluation is a very good predictor of culture-positive PJI (typical positive likelihood ratio >10) and is moderately accurate for ruling out this diagnosis (negative likelihood ratio, 0.2–0.3). In many hospitals, FS histopathology should now be considered a requisite part of the diagnostic workup for PJI, especially when the possibility of infection remains after thorough preoperative evaluation.

Nonetheless, the optimal diagnostic thresholds of PMNs per HPF on FS among studies are inconsistent. While Mirra et al. defined 5 PMNs per HPF as the gold standard [11], many studies have reported 5 PMNs per HPF as the optimal diagnostic threshold. Using the criterion of 5 PMNs per HPF, an independent study by Spangehl et al. [20] demonstrated a sensitivity of 80% and a specificity of 94%, while another study, by Wong et al. [21], concluded a sensitivity of 93% and a specificity of 77%. On the other hand, 2 recent meta-analyses [7], [22] determined that, compared with 5 PMNs per HPF, 10 PMNs is better for diagnosing PJI. Analysis of variance showed that the specificity was significantly higher in studies using 10 PMNs compared with 5 per HPF (*p* = 0.007), while an ROC curve confirmed the trend of superiority for 10 compared with 5 PMNs as the threshold [22]. However, owing to differences in PJI diagnostic criteria and patient characteristics among the studies included in the meta-analyses, the heterogeneity was significant. Our results support 5 PMNs per HPF as the optimal diagnostic threshold, whether in single- or multiplex-site FS.

In the present study, we found that single-site FS was not both highly sensitive and specific. Thus, we conducted an analysis of multiplex-site FS, including 3, 5, and 7 sites. In every subgroup analysis, 5 PMNs per HPF had the highest accuracy compared with 2 and 10 PMNs. For any 1 positive site in multiplex-site FS, as the number of sites increased, the sensitivity also increased but the specificity decreased. For every 1 positive site, as the number of sites increased, the sensitivity decreased but the specificity increased. We found that 5 PMNs per HPF in any 1 of 5 sites is a suitable diagnostic threshold, with a sensitivity of 0.86 and a specificity of 0.96.

### Utility of CRP Level, ESR, and Combined Test

Although multiplex tests for the perioperative identification of deep PJI are currently available, the ideal test should be sensitive and specific as well as cost-effective, readily available, and simple to perform. The use of preoperative CRP level and ESR for routine screening before revision total joint arthroplasty has been advocated because these tests are easily obtained and reported to have considerable sensitivity for identifying PJI [15]–[17], [23]–[27]. Because single-site FS is not both highly sensitive and specific, 2 tests are often combined to potentially raise the sensitivity and/or specificity. Our results suggest that simultaneous administration of both tests (such as “5 PMNs in single-site FS OR CRP level ≥15 mg/L”), with a positive result in at least 1 test considered an indication of PJI, increased the sensitivity to 0.92. The sensitivity of the combined tests was significantly higher than that of either test alone. However, combining the 2 tests decreased the specificity to 0.79. The ideal screening test should be highly sensitive, and the apparent increase in sensitivity indicates that this combination is good for ruling out PJI. The results show that when PMNs and CRP level are measured together as a screening battery (with a positive result in either test defined as a positive result), the negative predictive value is 0.99. In other words, if both tests are negative, then there is scant probability that PJI is present in a symptomatic joint arthroplasty patient.

In contrast, tests with a high specificity are used to confirm the presence of disease. Therefore, if we desire better specificity in a diagnostic test battery, the combination of PMNs and CRP level both positive (i.e., “5 PMNs in single-site FS AND CRP level ≥15 mg/L”; specificity, 0.99; sensitivity, 0.65; positive predictive value, 0.96) is preferred. The specificity of this combination is higher than that of either test alone. In our symptomatic joint arthroplasty patients, the posttest probability of infection was 96% when both PMNs and CRP level were positive.

### Strengths and Limitations of This Study

Our study has several strengths over previous studies on this topic. First, to the best of our knowledge, this is the first study to investigate the utility of intraoperative FS in the diagnosis of PJI in a Chinese population. Compared with whites, Chinese individuals more frequently use antibiotics when they have a common cold or other diseases. Therefore, we believe it is very significant to investigate the diagnostic method for infection in the Chinese population. Second, few studies have evaluated the diagnostic utility of multiplex-site FS and combined tests with inflammatory laboratory markers. BY investigating the utility of multiplex-site FS and combined tests, our study contributes to the building of a highly effective diagnostic method to identify PJI.

Nevertheless, this study has some limitations. First, although the diagnostic criteria for PJI were agreed upon by surgeons and pathologists, interpathologist differences in interpretation could have biased the results. The reproducibility of results vary among pathologists and even with the same pathologist, regardless of their training and experience. The variation identified is nearly10%. Second, the number of patients with PJI in this study was rather small, which may have reduced the generalizability of our conclusions. Finally, the small number of patients who underwent FS of 7 sites in our study may have reduced the reliability of our conclusions.

### Conclusion

On the basis of these data, compared with single-site FS, any 1 of multiplex-site FS may improve sensitivity and every 1 of multiplex-site FS may improve specificity. Five PMNs in any 1 of 5 sites has excellent utility for the diagnosis of PJI. Additional systematic large-scale studies are needed to verify this result.

## Supporting Information

Figure S1 **The flow diagram for PMN = 2/5/10 on single site intraoperative frozen section.**
(PDF)

Checklist S1 **STARD checklist for reporting of studies of diagnostic accuracy (version January 2003).**
(DOC)
