# Supplementary material for: Utility of Intraoperative Frozen Section in the Diagnosis of Periprosthetic Joint Infection
Source: PLoS One. 2014 Jul 15;9(7):e102346. doi: 10.1371/journal.pone.0102346 (PMC4099375; doi:10.1371/journal.pone.0102346)

Flow diagram for PMN=2 on Single site FS

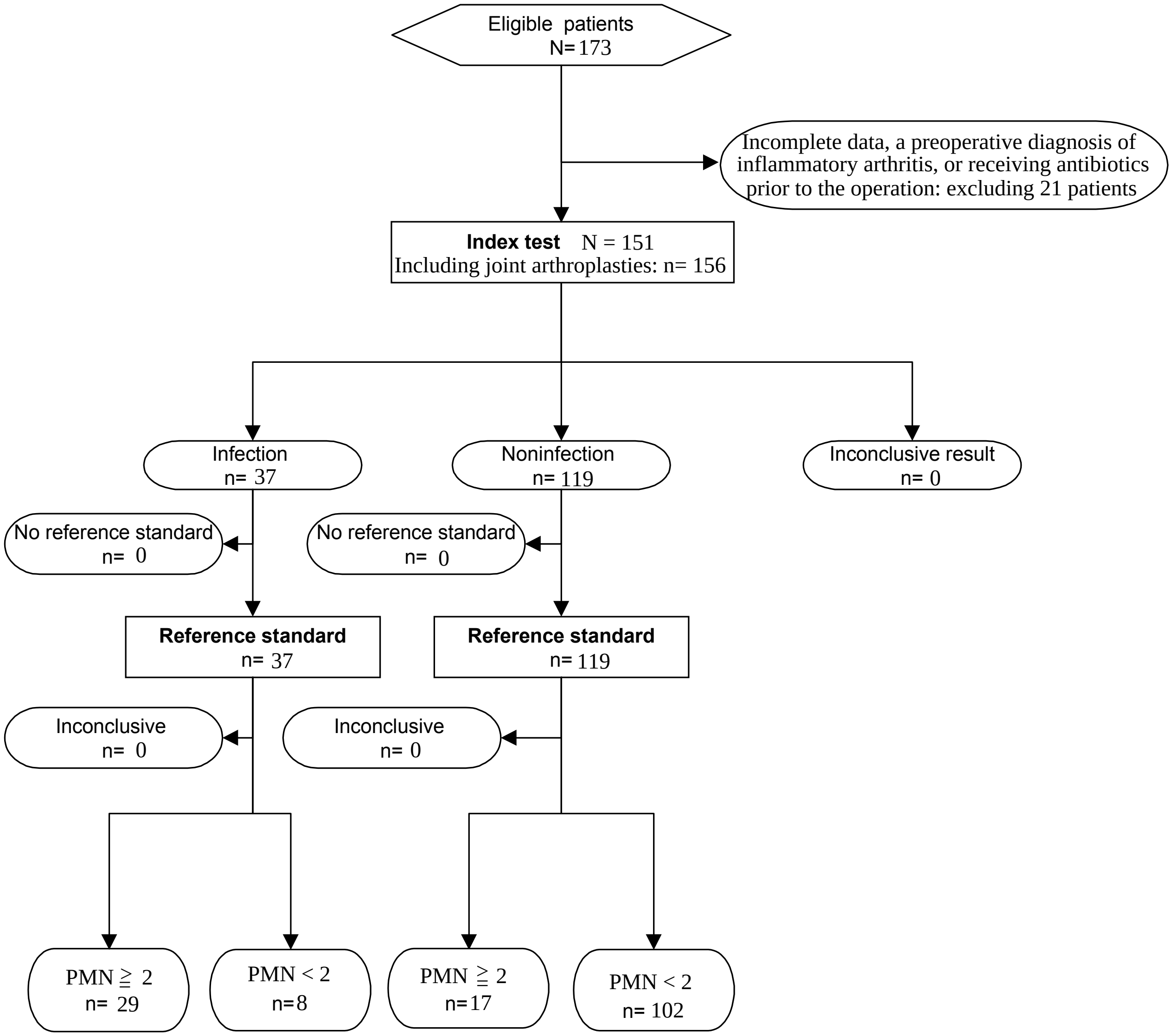

Flow diagram for PMN=5 on Single site FS

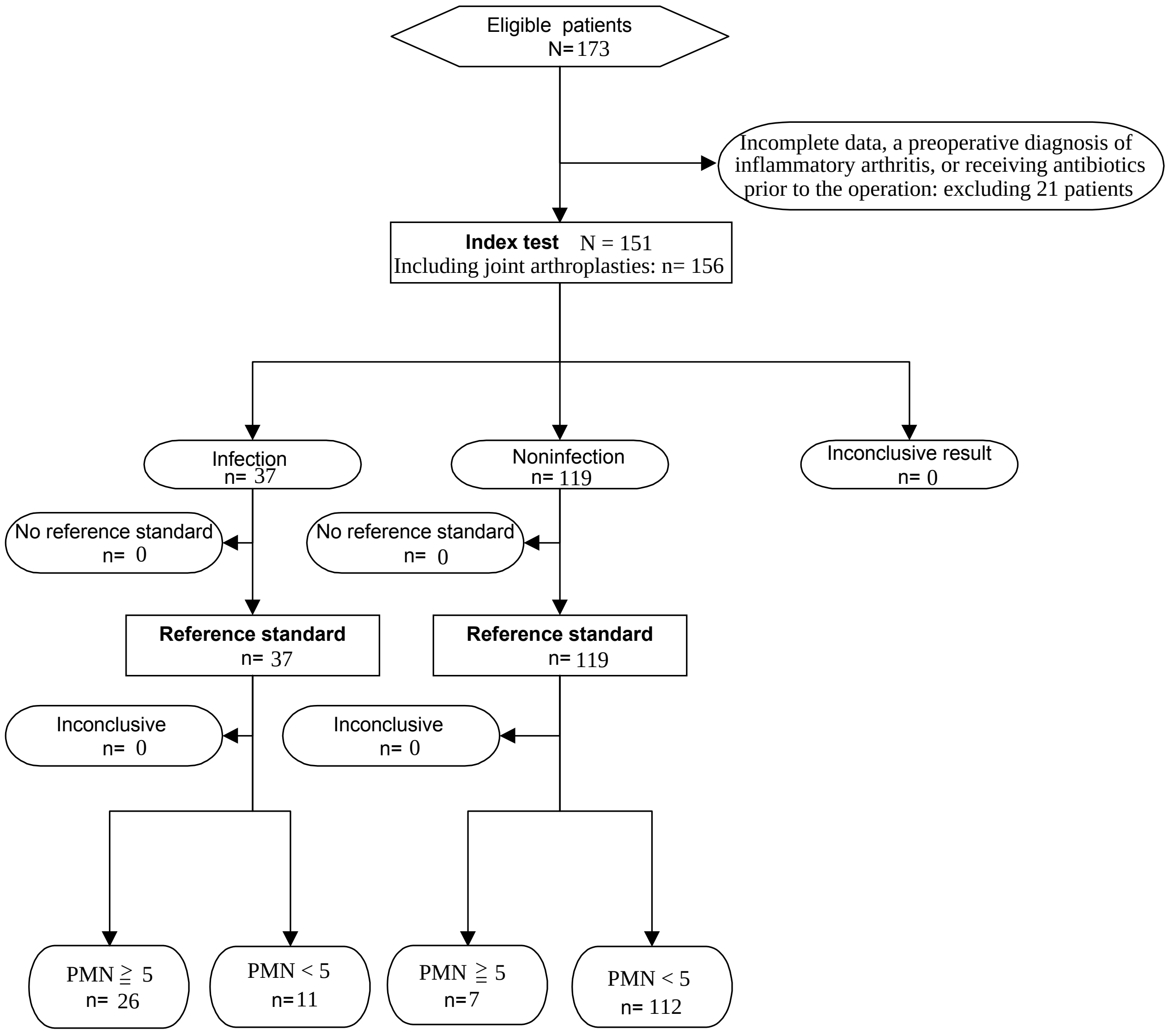

Flow diagram for PMN=10 on Single site FS

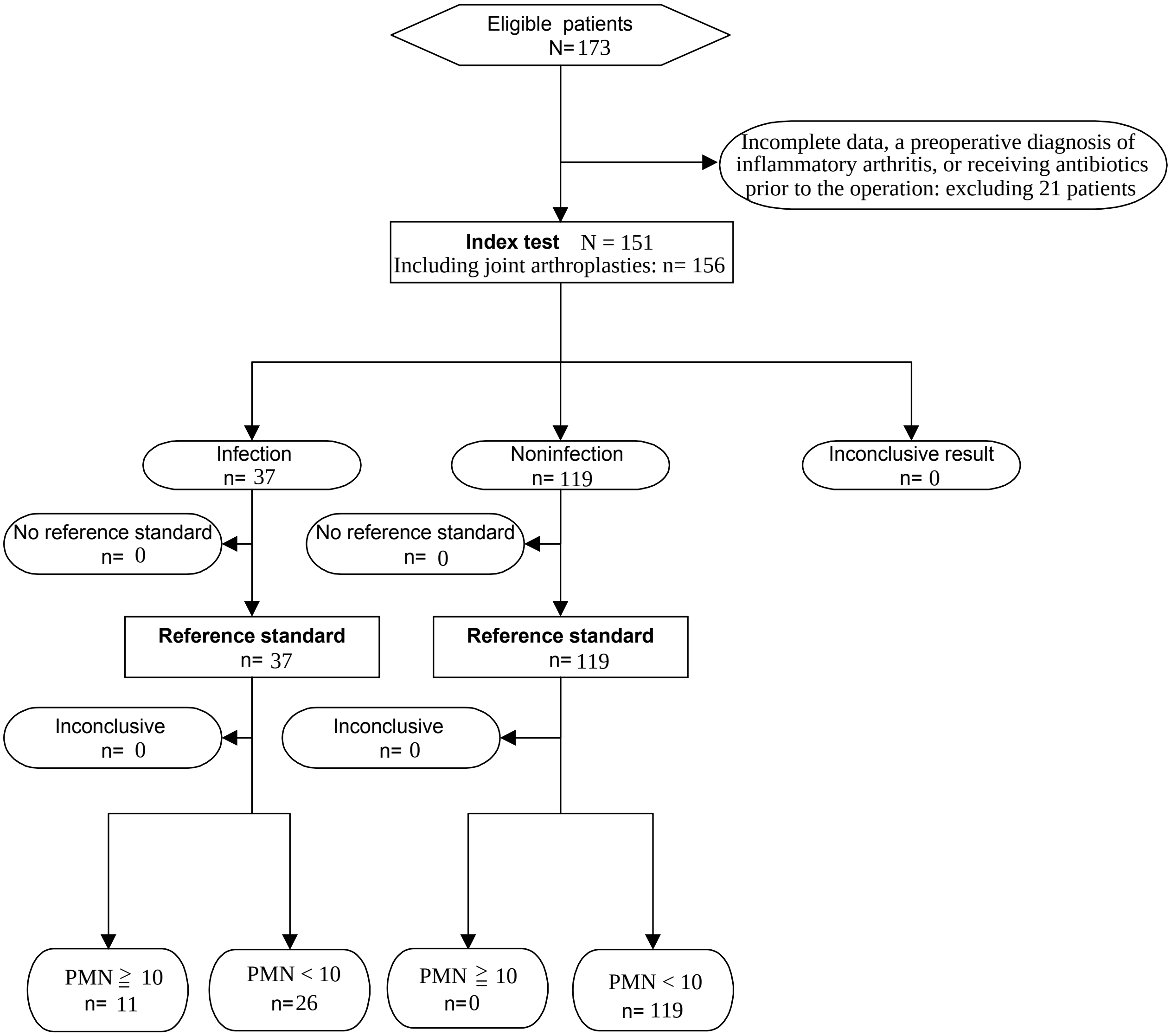

Supplement: Figure S1 — The flow diagram for PMN = 2/5/10 on single site intraoperative frozen section. (PDF) [file pone.0102346.s001.pdf]
